# Added Value Measures in Education Show Genetic as Well as Environmental Influence

**DOI:** 10.1371/journal.pone.0016006

**Published:** 2011-02-02

**Authors:** Claire M. A. Haworth, Kathryn Asbury, Philip S. Dale, Robert Plomin

**Affiliations:** 1 Social, Genetic and Developmental Psychiatry Centre, King's College London, London, United Kingdom; 2 Department of Speech and Hearing Sciences, University of New Mexico, Albuquerque, New Mexico, United States of America; University of Oxford, United Kingdom

## Abstract

Does achievement independent of ability or previous attainment provide a purer measure of the added value of school? In a study of 4000 pairs of 12-year-old twins in the UK, we measured achievement with year-long teacher assessments as well as tests. Raw achievement shows moderate heritability (about 50%) and modest shared environmental influences (25%). Unexpectedly, we show that for indices of the added value of school, genetic influences remain moderate (around 50%), and the shared (school) environment is less important (about 12%). The pervasiveness of genetic influence in how and how much children learn is compatible with an active view of learning in which children create their own educational experiences in part on the basis of their genetic propensities.

## Introduction

A measure of academic achievement uncorrelated with ability or previous attainment is an appealing construct, relevant for several current controversies and goals in education [1], [2], [3]. Such a measure, conceptualized as reflecting environmental influence [4], might serve as an index of ‘added value’ provided by schools or teachers for purposes of evaluation. A novel approach in understanding measures of added value is to assess the joint and independent contributions of nature (genetics) and nurture (environment) to added value indicators. To be able to tease apart the effects of nature and nurture, a genetically sensitive study is needed, such as a twin or adoption study [5]. To date, these methods have been used sparingly in educational research [6], although they have been used widely in psychological and medical research with great success [7]. In the current paper we investigate the issue of ‘added value’ using a twin design. We assess the extent to which academic achievement measures that are corrected for previous attainment (both general cognitive ability and previous school performance) are ‘purer’ measures of the environmentally driven ‘added value’.

### The Twin Method

Twin studies provide a useful indication of the relative contributions of genetic and environmental factors on individual differences in measured traits [5], [7], [8], [9]. The twin method uses MZ (monozygotic, identical) and DZ (dizygotic, fraternal) twin intraclass correlations to dissect phenotypic variance into genetic and environmental sources [7]. MZ twins are genetically identical in terms of inherited variation in DNA sequence, whereas DZ twins are on average only 50% similar for segregating alleles. Environmental variance can be dissected into shared environmental effects (i.e., environmental effects that make members of the same family more similar) and non-shared environmental effects (i.e., environmental effects that do not make members of the same family similar). These genetic and environmental effects are commonly known as A, C and E. ‘A’ is the additive genetic effect size, also known as narrow heritability. Heritability can be estimated by doubling the difference between MZ and DZ twin correlations. Shared environment (C, for effects in common to family members) refers to variance that makes MZ and DZ twins similar beyond twin similarity explained by additive genetic effects. C can be estimated by subtracting the estimate of heritability from the MZ correlation. In addition, non-shared environmental influences (E) can be estimated from the total variance not shared by MZ twins; non-shared environmental influences are the only influences deemed to make MZ twins different. E also includes measurement error. A more elegant way of estimating the ACE parameters is maximum likelihood structural equation model fitting analysis [7], [9], which provides more detailed estimates of genetic and environmental effect sizes that make assumptions explicit and provides confidence intervals for the parameter estimates.

Behavioral genetics and the twin method are concerned with the genetic and environmental influences on individual differences in complex traits. Such complex traits are the outcome of multiple genetic factors alongside (and potentially in interaction with) multiple environmental factors throughout development. The focus is on variation in genes and environments in relation to variation in outcomes; as such the statistics of behavioral genetics concern population (or sample) level effects, rather than case studies of particular individuals' genetic and environmental profiles.

Previous twin studies on school performance have indicated moderate heritability around 40–60% [10], [11], [12], [13]. Studies specifically focusing on reading abilities show a similar pattern of results with moderate to high heritability and modest shared environmental influences [14], [15]. More recently, studies have also included mathematical abilities, which typically show high heritabilities around 60–70% [16], [17]. A striking finding is that genetic influences appear to have largely generalist effects across diverse cognitive and academic abilities [18], [19]. For example, the average genetic correlation (an index of the degree to which genetic influences on one trait also influence another trait) between diverse cognitive and academic domains was 0.70 in a recent review [19]. At first glance, this high degree of genetic overlap between different cognitive and academic measures suggests that correcting achievement measures for general cognitive ability would remove the genetic influence on achievement. However, this genetic overlap is not 100%, so there could be residual genetic influences on achievement that are independent of those on general cognitive ability, or even previous measures of achievement.

### Evaluating schools

The evaluation of schools has increased steadily since the early 1990s, with the ultimate aim of encouraging competition and thereby driving improvements in education. The concept of ‘added value’ was introduced to the UK school system in 2002 to overcome the issues of using ‘raw’ school attainment as a measure of school performance, because raw achievement is largely an index of the calibre of pupil intake, rather than any indication of the school's performance [1], [20]. The concept is straightforward: the notion is to estimate the additional knowledge gained over a certain period of schooling by controlling for previous attainment. Taylor and Nguyen [21] state that:

*“The inclusion of the previous level of attainment is therefore intended to capture the effects on the current level of knowledge of all historical inputs, including inherited endowments such as innate ability as well as family background and schooling.”* (page 209).

Education has been slow to accept the role of genetic influences on educational outcomes [6]; yet in the statement above, added value has been linked with the removal of ‘innate ability’. The current study aims to investigate whether removing ability and previous attainment from school achievement does in fact remove the genetic influence on school performance. One concern involves the choice of measure to use to control for ‘previous attainment’. Within the literature some studies have used cognitive abilities tests as their measure of previous attainment [3], whereas others have focused on assessments of previous school performance [22]. For this reason, we conduct analyses using both ability (i.e. general cognitive ability, or intelligence) and previous school achievement measures. In addition, we extend these analyses by controlling for both ability and previous achievement, which provides a strong test of any residual genetic influence on school achievement outcomes.

Analyses of the determinants of added value suggest that both school-level and pupil-level characteristics are important [4], [21]. Given that the concept of added value is to assess the contribution of the school environment to students' achievement, it seems reasonable to predict that indices of added value will show more shared environmental influence than raw measures of achievement, and that genetic influences will be reduced. However, from our genetic perspective, we would be surprised to see the complete removal of genetic influence, so we would therefore predict that school achievement will remain moderately heritable even after controlling for ‘innate ability’ or previous achievement.

## Methods

### Sample

The sampling frame for the present study was the Twins Early Development Study (TEDS), a study of twins born in England and Wales in 1994, 1995, and 1996 [23], [24]. The TEDS sample has been shown to be reasonably representative of the general population in terms of parental education, ethnicity and employment status [16]. Zygosity was assessed through a parent questionnaire of physical similarity, which has been shown to be over 95% accurate when compared to DNA testing [25]. For cases where zygosity was unclear from this questionnaire, DNA testing was conducted. At the time of the ‘Age 12’ assessment, the mean age of the twins taking part in the study was 11.54 (sd  = .66). 6090 families (73.9% of those contacted) took part in the web-based testing, and we received teacher questionnaires for 9906 individuals, including 4405 complete twin pairs (78.1% of those sent). Not all teachers provided information on National Curriculum levels and some twins did not complete all of the web tasks; exact N values for our measures of ability and achievement are presented below. Ethical approval for the Twins Early Development Study has been provided by the King's College London ethics committee (reference: 05/Q0706/228). The parents of the twins provide informed written consent for each TEDS assessment.

### Measures

#### Teacher-rated achievement

The twins' academic performance was assessed throughout the school year by their teachers, using the assessment materials of the National Curriculum for England and Wales (NC), the core academic curriculum developed by the Qualifications and Curriculum Authority (QCA) [26]. Teachers were contacted when the children were in the second half of their school year so that the teachers would be familiar with the children's performance during the school year. Teachers were sent a covering letter with the background and aims of TEDS, as well as explaining that we had obtained consent from the twins' parents to ask teachers for information about the child's performance at school.

For the study at age 12, the NC Teacher Assessments at Key Stage 3 were used, which are designed for children aged 11–14 years old. When the twins were 12 years old, teachers assessed three broad areas of achievement: English (including speaking and listening; reading; and writing); mathematics (including using and applying mathematics; number and algebra; shape, space and measures; and handling data); and science (including scientific enquiry; life processes and living things; physical processes; and materials and their properties). Teachers rate performance from levels 1 to 8. By the end of Key Stage 3 at age 14, children are expected to reach levels 5/6. These judgments were not made specifically for the present study, but rather form the continuing assessment of each child that ultimately leads to the final NC Teacher Assessment score submitted to the QCA at the end of the key stage, which summarizes the child's academic achievement during that period. Reminders of the NC criteria used to select the appropriate attainment level were provided as part of the questionnaire.

The teacher-rated achievement scales are highly correlated, with an average intercorrelation of .83. A factor analysis indicated that the first principal component accounted for 89% of the variance. For this reason, we calculated a composite achievement score as a mean of the three achievement domains: English, mathematics and science. There were 2824 complete pairs of twins with this NC achievement composite; 1039 monozygotic (MZ, identical) pairs; and 1785 dizygotic (DZ, fraternal) pairs. Further details about these measures have been published previously [16], [18], and further information about the UK National Curriculum is available at http://curriculum.qca.org.uk.

#### Tests of achievement

To complement our teacher ratings of school achievement we also collected test data on reading and math performance at 12 years. We created a web-based cognitive battery to allow collection of test data from the whole TEDS sample, which is spread across the UK. We have shown that our web-based cognitive test battery is a reliable and valid method for collecting cognitive data on children as young as 10 years old [27]. For example our mathematics web battery and the equivalent paper-and pencil test correlated .92 [27]. More than 80% of the TEDS sample has access to the internet at home and most children without access to the internet at home have access in their schools and local libraries. Additionally, we found no correlation between internet speed (i.e. broadband versus dial-up connection) and socio-economic status (SES). At 12 years we included three measures of reading achievement and three measures of mathematics achievement in our cognitive web battery.

#### Reading

Three measures of reading ability were used at 12 years: two measures of reading comprehension and a measure of reading fluency. The twins completed an adaptation of the reading comprehension subtest of the Peabody Individual Achievement Test [28], which we will refer to as PIAT_rc_. The PIAT_rc_ assesses literal comprehension of sentences. The sentences were presented individually on the computer screen. Children were required to read each sentence and were then shown four pictures. They had to select, using the mouse, the picture that best matched the sentence they had read. All children started with the same items, but an adaptive algorithm modified item order and test discontinuation depending on the performance of the participant. The internet-based adaptation of the PIAT_rc_ contained the same practice items, test items and instructions as the original published test.

As well as the PIAT_rc_, we assessed reading comprehension at age 12 using the GOAL Formative Assessment in Literacy for Key Stage 3 [29]. The GOAL is a test of reading achievement that is linked to the literacy goals for children at Key Stage 3 of the National Curriculum. Questions are grouped into three categories: Assessing Knowledge and Understanding (e.g., identifying information, use of punctuation and syntax), Comprehension (e.g., grasping meaning, predicting consequences), and Evaluation and Analysis (e.g., comparing and discriminating between ideas). Within each category, questions about words, sentences, and short paragraphs are asked. Because we were primarily interested in comprehension skills, we used questions from the two relevant categories, Comprehension, and Evaluation and Analysis (20 items from each category). Correct answers were summed to give a total comprehension score.

Reading fluency was assessed using an adaptation of the Woodcock-Johnson III Reading Fluency Test [30]. This is a measure of reading speed and rate that requires the ability to read and comprehend simple sentences quickly e.g. “A flower grows in the sky? - Yes/No”. Low performance on reading fluency may be a function of limited basic reading skills or comprehension. The online adaptation consists of 98 yes/no statements; children need to indicate yes or no for each statement, as quickly as possible. There is a time limit of 3 minutes for this test. Correct answers were summed to give a total fluency score.

#### Mathematics

In order to assess mathematics, we developed an internet-based battery that included questions from three different components of mathematics. The items were based on the National Foundation for Educational Research 5-14 Mathematics Series, which is linked closely to curriculum requirements in the UK and the English Numeracy Strategy [31]. The presentation of items was streamed, so that items from different categories were mixed, but the data recording and branching were done within each category. The items were drawn from the following three categories: Understanding Number, Non-Numerical Processes and Computation and Knowledge. The mathematics battery is described in more detail elsewhere [32].

The test achievement scales were moderately correlated, with an average intercorrelation of .49. A factor analysis indicated that the first principal component accounted for 58% of the variance. We calculated a composite achievement score as the mean of the six achievement web measures. There were 5142 pairs of twins with this web achievement composite; 1892 MZ; and 3250 DZ.

#### Tests of ability

As well as tests of achievement, we also included ability tests in our web-based cognitive battery at 12 years. The twins were tested on two verbal tests, WISC-III-PI Multiple Choice Information (General Knowledge) and Vocabulary Multiple Choice subtests [33], and two non-verbal reasoning tests, the WISC-III-UK Picture Completion [33], and Raven's Standard and Advanced Progressive Matrices [34], [35]. We created a general cognitive ability (*g)* score as the mean of the four tests.

#### Measures at 10 years

When we correct for previous achievement at 10 years we use the same NC teacher reports (English, mathematics and science) and tests (reading (PIAT_rc_) and mathematics (NferNelson) from our 10-year web-battery [27].

### Analyses

All measures were standardized to a mean of zero and a standard deviation of 1 on the basis of the entire sample of twins (with children with major perinatal and medical problems excluded). To create the ability-corrected achievement scores, both teacher ratings and test-based scores, we used a standard regression onto general cognitive ability, and saved the standardized residuals. The correlations between *g* and achievement for teacher ratings and test data before the regression were .50 and .69; and after the regression both were .00. Thus our corrected achievement scores do not correlate with general cognitive ability. These results are highly similar to those found in research using more conventional measures; in elementary school correlations of about 0.45 between teacher assessments of achievement and *g* are typically found, and correlations of about 0.65 between test scores of achievement and *g* [36]. The same regression procedure was used to correct for previous achievement, as well as the combined effect of previous achievement and ability (g).

Because twins are perfectly correlated for age and same-sex twins are correlated perfectly for sex, variation associated with age or sex would contribute to the correlation between twins. That is, data uncorrected for age and sex would inflate twin correlations. For this reason, and as is standard in twin analysis, all measures were also corrected for age and sex effects using a regression procedure [37].

Twin intraclass correlations were calculated, which index the proportion of total variance due to between-pair variance [38]. Rough estimates of genetic (A), shared environmental influences (C; that makes twin pairs more similar to one another), and non-shared environmental influences (E; that no not contribute to similarity between twins), can be calculated from these twin correlations. Mx software for structural equation modelling was used to perform standard twin model-fitting analyses [39].

### Multivariate twin analysis

A more elegant way to address the question of “*g-*free achievement” (or achievement adjusted for previous achievement) than residualizing on *g,* is multivariate genetic analysis, which estimates the extent to which genetic and environmental factors that affect one trait also affect another trait. Rather than removing the *g* covariance from the variance of achievement scores prior to analysis, multivariate genetic analysis considers all of the variance of achievement scores as well as all of the variance of g, and it decomposes the covariance between them into genetic and environmental components of covariance [7], [40]. In other words, multivariate twin analysis uses the twin method to estimate genetic and environmental contributions to the covariance of two or more traits as well as the variance of each trait [39]. For the bivariate analysis between general cognitive ability and achievement we used the standard Cholesky decomposition model, which tests for common and independent genetic and environmental effects on variance in different traits. The Cholesky procedure is similar to hierarchical regression analyses in non-genetic studies, where the independent contribution of a predictor variable is assessed after accounting for its shared variance with other predictor variables. In this case, the first factor assesses genetic, shared and non-shared environmental influences on g, some of which may also influence school achievement. The second factor estimates influences on achievement that are independent of the influences on g. The same bivariate Cholesky approach was used to estimate genetic and environmental influences on achievement at age 12 that are independent of previous achievement. A trivariate Cholesky decomposition model was used to estimate the influences on 12-year achievement that are independent of those on both previous achievement and g.

## Results

The means and standard deviations (*SD*) for the measures at 10 and 12 years are presented in **Table 1**. ANOVA was used to assess the effects of sex and zygosity on each of our measures. We used standardized measures for these analyses, and the ANOVA was performed before age and sex regression. The results of a 2×2 (sex by zygosity) ANOVA, shown in **Table 2**, indicate no significant effects (p<.01) of sex on achievement scores. Small, but significant (p<.001) effects of sex were found for general cognitive ability. There were significant main effects (p<.01) of zygosity on 12-year test achievement and general cognitive ability, but these significant effects are very small, generally accounting for less than 1% of the variance, and are likely due to the large sample size. There were no significant interactions between sex and zygosity. The raw National Curriculum levels, as reported by teachers, are consistent with the expected level of achievement for this age group (e.g., at 12 years: English mean = 4.35; s.d. = 0.965; Maths mean = 4.38; s.d. = 1.03; Science mean = 4.44; s.d. = 0.953).

**Table 1:** Means (and standard deviations), by zygosity and sex.

Age	Measure	All	MZ	DZ	Female	Male
10	Teacher-rated Achievement	0.00 (1.01) N = 2699	−0.04 (1.02) N = 958	0.03 (1.00) N = 1741	0.02 (0.97) N = 1425	−0.01 (1.05) N = 1274
	Test Achievement	0.00 (0.99) N = 2926	−0.05 (1.00) N = 1058	0.03 (0.98) N = 1868	−0.04 (0.98) N = 1601	0.06 (1.00) N = 1325
12	Teacher-rated Achievement	0.01 (1.01) N = 3568	−0.03 (1.00) N = 1284	0.03 (1.02) N = 2284	0.02 (0.98) N = 1885	0.00 (1.05) N = 1683
	Test Achievement	0.01 (1.00) N = 5366	−0.05 (1.00) N = 1955	0.04 (1.00) N = 3411	−0.01 (0.98) N = 2906	0.03 (1.02) N = 2460
	General Cognitive Ability	0.00 (1.00) N = 4235	−0.07 (0.99) N = 1575	0.04 (1.00) N = 2660	−0.06 (0.99) N = 2366	0.07 (1.00) N = 1869

*Note.* MZ  =  monozygotic; DZ  =  dizygotic; means for one randomly selected member of each twin pair (N indicates number of randomly selected individuals).

**Table 2:** ANOVA results showing significance and effect size, by zygosity and sex.

Age	Measure	Zygosity	Sex	Zygosity * sex
10	Teacher-rated Achievement	*p* = .125 *η^2^* = 0.001	*P* = .454 *η^2^*<0.001	*p* = .726 *η^2^*<0.001
	Test Achievement	*p* = .037 *η^2^* = 0.001	*P* = 012 *η^2^* = 0.002	*p* = .938 *η^2^*<0.001
12	Teacher-rated Achievement	*p* = .057 *η^2^* = 0.001	*p* = .489 *η^2^*<0.001	*p* = .381 *η^2^*<0.001
	Test Achievement	*p* = .001 *η^2^* = 0.002	*p* = .167 *η^2^*<0.001	*p* = .394 *η^2^*<0.001
	General Cognitive Ability	*p* = .003 *η^2^* = 0.002	*p*<.001 *η^2^* = 0.004	*p* = .129 *η^2^* = 0.001

*Note. η^2^* =  eta squared (effect size). ANOVA for one randomly selected member of each twin pair.

### Intraclass twin correlations

The twin intraclass correlations are shown in **Table 3** for MZ and DZ twins. Are our indices of added value less heritable than raw achievement measures? For all measures, MZ correlations exceeded those of the DZ twins, suggesting genetic influence. For the entire sample, doubling the difference between the MZ and the DZ correlations to estimate heritability indicated substantial heritability (.44) for g-free teacher-rated achievement at age 12, only slightly less than for raw teacher-rated achievement at age 12 (.50). For g-free tested achievement, heritability was also substantial (.40), only somewhat less than heritability for raw tested achievement (.56). Similar results emerged for 12-year achievement corrected for achievement at age 10: Heritability for 12-year achievement independent of 10-year achievement was .44 for teacher ratings and .52 for test data.

**Table 3:** Intraclass twin correlations for 12-year achievement, general cognitive ability (g), and for achievement corrected for g and achievement corrected for previous achievement.

Measure	rMZ	rDZ
Teacher-rated Achievement	0.80 N = 1039	0.55 N = 1785
Test Achievement	0.75 N = 1892	0.47 N = 3250
General Cognitive Ability (*g*)	0.67 N = 1523	0.45 N = 2527
g-free Teacher-rated Achievement	0.67 N = 681	0.45 N = 1112
g-free Test Achievement	0.46 N = 1478	0.26 N = 2461
Teacher-rated Achievement (corrected for 10y Ach)	0.57 N = 185	0.35 N = 326
Test Achievement (corrected for 10y Ach)	0.56 N = 733	0.30 N = 1242

*Note.* rMZ  =  monozygotic twin correlation; rDZ  =  dizygotic twin correlation; N indicates number of complete twin pairs; 10y Ach  =  10-year achievement.

Do our indices of added value show greater influence of shared (school) environments? Shared environment can be estimated by subtracting the above estimates of heritability from the MZ twin correlation. Shared environment was actually *lower* when achievement was independent of g: .23 versus .30 for teacher-rated achievement and .06 versus .19 for tested achievement. Similar results were found when 12-year achievement was corrected for achievement at age 10: Shared environment estimates were .13 for teacher-rated achievement and .04 for tested achievement. The remainder of the variance is attributed to non-shared environmental influences, which include measurement error. This component of variance was substantially greater when achievement was corrected for g: .33 versus .20 for teacher ratings and .54 versus .25 for test scores.

### Univariate model-fitting analyses of raw achievement and corrected achievement

Structural equation maximum likelihood model-fitting analyses were performed in the program Mx [39] using the standard twin model. This model decomposes the variance into genetic (A), shared environmental (C) and non-shared environmental (E) components. Model-fitting ACE estimates are included in **Table 4** (with 95% confidence intervals), and are similar to the simple estimates derived from the twin intraclass correlations. For example, heritabilities were 0.52 for teacher-rated achievement, 0.49 for g-free teacher-rated achievement, and 0.47 for 12-year teacher-rated achievement corrected for 10-year achievement. These are non-significant differences as indicated by the overlapping confidence intervals for these heritability estimates.

**Table 4:** ACE model-fitting estimates (95% CIs) for 12-year achievement, general cognitive ability (g), and for achievement corrected for g and achievement corrected for previous achievement.

Measure	A	C	E
Teacher-rated Achievement	0.52 (.48–.57)	0.30 (.26–.35)	0.17 (.17–.18)
Test Achievement	0.55 (.50–.61)	0.20 (.20–.25)	0.25 (.23–.27)
General Cognitive Ability (*g*)	0.47 (.42–.52)	0.20 (.16–.25)	0.33 (.32–.34)
g-free Teacher-rated Achievement	0.49 (.41–.56)	0.21 (.14–.27)	0.31 (.28–.33)
g-free Test Achievement	0.40 (.29–.49)	0.06 NS (.00–.15)	0.54 (.50–.58)
Teacher-rated Achievement (corrected for 10y Ach)	0.47 (.21–.66)	0.12 NS (.00–.32)	0.41 (.33–.51)
Test Achievement (corrected for 10y Ach)	0.46 (.33–.58)	0.08 NS (.00–.19)	0.46 (.41–.50)

*Note.* A =  Additive genetic; C =  Shared environment; E =  Non-shared environment; NS  =  Non-significant at 0.05 alpha level; 10y Ach  =  10-year achievement.

As indicated by the pattern of MZ and DZ twin correlations, shared environment was *lower* when achievement was corrected for g: .30 versus .21 for teacher-rated achievement, a non-significant difference, and .20 versus .06 for tested achievement, a significant difference. And similar results were found for achievement corrected for previous achievement: .30 versus .12 for teacher-rated achievement (non-significant difference), and .20 versus .08 for test achievement (significant difference). Non-shared environmental influences were significantly greater when achievement was corrected for either g or for previous achievement.

### Bivariate model-fitting analyses

**Figure 1:**
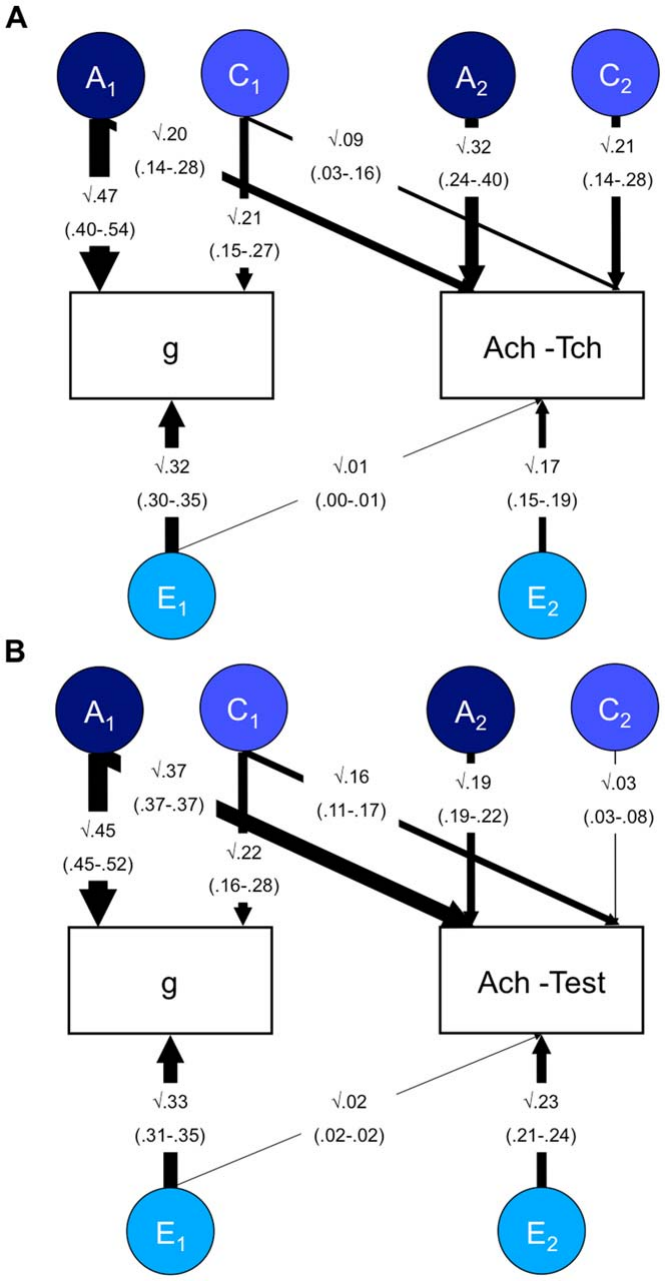
Bivariate analysis of *g* and achievement. Panel A = g and teacher-rated achievement at 12 years. Panel B = g and test achievement at 12 years. g =  general cognitive ability; Ach-Tch  =  Teacher-rated achievement; Ach-Test  =  test achievement; A =  additive genetic; C =  Shared environment; E =  Non-shared environment. The figures represent the results from a standardized Cholesky decomposition of twin data. 95% confidence intervals of the path estimates are shown in parentheses. The first factors assess genetic (A_1_), shared (C_1_) and non-shared environmental (E_1_) influences on *g*, some of which may also influence school achievement. The second factor estimates influences on achievement that are independent of the influences on *g* (A_2_, C_2_ and E_2_). Results indicate significant residual genetic influence on school achievement, even when the genetic and environmental co-variance with general cognitive ability has been removed (see the A_2_ path estimate).

Results from the bivariate Cholesky decomposition model for general cognitive ability and 12-year achievement are shown in **Figure 1a** for teacher-rated achievement, and in **Figure 1b** for test scores of achievement. The A_2_, C_2_ and E_2_ estimates indicate the genetic, shared environmental and non-shared environmental influences on achievement that are independent of those influences on g. For teacher-rated achievement these g-free estimates are .32, .21 and .17 respectively, all statistically significant. The total g-independent variance of teacher-rated achievement is .70 (i.e., .32+.21+.17 = .70). If we re-standardize the g-independent genetic and environmental components of variance on the basis of this total g-independent variance of .70, the resulting ACE estimates are similar to the univariate model-fitting results reported in **Table 4** for achievement phenotypically independent of g. That is, g-free heritability from **Figure 1a** is .46 (.32/.70 = .46), g-free shared environment is .30 (.21/.70 = .30), and g-free non-shared environment is .24 (.17/.70 = .24). In **Table 4** the comparable g-free ACE estimates are .49, .21, and .31.

A similar pattern is found for genetic and shared environmental influences on test data of achievement (**Figure 1b**). The significant g-free genetic estimate of .19 indicates that genetic factors independent of g can account for 19% of the total variance of tested achievement. g-free heritability is .42 (.19/.45 = .42), which is similar to the estimate of .40 using the regression procedure (**Table 4**). In contrast, the g-free shared environment estimate of .03 in **Figure 1b** is not significant, indicating that there is no significant shared environmental influence on tested achievement independent of g. Another way of expressing this finding is that shared environment only accounts for 7% of the variance of g-free tested achievement (.03/.45 = .07), similar to the estimate of 6% in Table 4 using the regression procedure.

**Figure 2:**
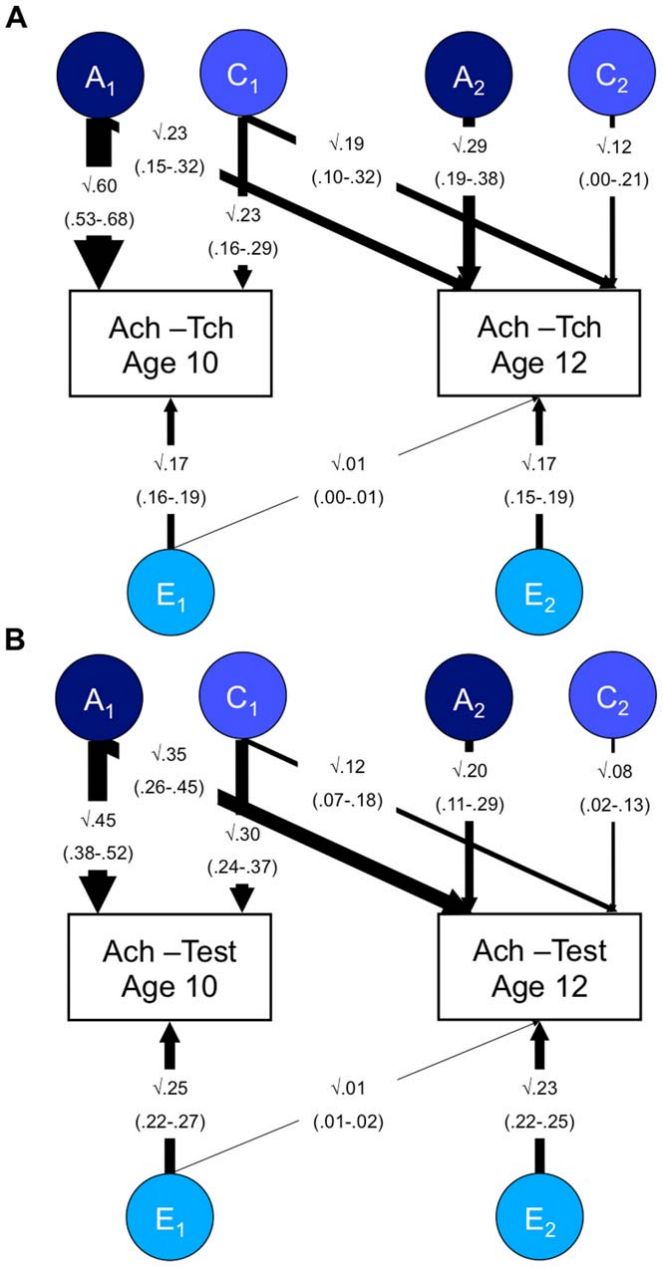
Bivariate analysis of 10 and 12-year achievement. Panel A =  Teacher-rated achievement at 10 and 12 years. Panel B =  Test achievement at 10 and 12 years. Ach-Tch  =  Teacher-rated achievement; Ach-Test  =  test achievement; A =  additive genetic; C =  Shared environment; E =  Non-shared environment. The figures represent the results from a standardized Cholesky decomposition of twin data. 95% confidence intervals of the path estimates are shown in parentheses. The first factors assess genetic (A_1_), shared (C_1_) and non-shared environmental (E_1_) influences on 10-year achievement, some of which may also influence 12- year school achievement. The second factor estimates influences on 12-year achievement that are independent of the influences on 10-year achievement (A_2_, C_2_ and E_2_). Results indicate significant residual genetic influence on 12-year school achievement, even when the genetic and environmental co-variance with previous achievement has been removed (see the A_2_ path estimate).

Results from the bivariate Cholesky decomposition model for 10 and 12-year achievement are shown in **Figure 2** for both teacher-rated achievement at 10 and 12 years (**Figure 2a**), and test scores of achievement at 10 and 12 years (**Figure 2b**). In **Figure 2a**, the significant A_2_ estimate of .29 indicates that 29% of the total variance of teacher-rated achievement at age 12 can be attributed to genetic influences independent of teacher-rated achievement at age 10. Similar to the results for g-free achievement, we can re-standardize the influences on 12-year achievement that are independent of those on 10-year achievement (the A_2_, C_2_ and E_2_ parameters). For teacher-rated achievement, the total variance of 12-year achievement that is independent of 10-year achievement is .58 (i.e. .29+ .12+ .17 = .58), so the re-standardized heritability of 12-year achievement independent of 10-year achievement is 50% (.29/.58 = .50). The shared environmental estimate (.12) is not significant, indicating that shared environment independent of 10-year achievement does not contribute significantly to 12-year achievement; the re-standardized shared environmental component is 21% (.12/.58 = .21). Results for test scores of achievement at ages 10 and 12 (**Figure 2b**) are similar to those of teacher-rated achievement: The significant A_2_ estimate of .20 indicates that 20% of the total variance of tested achievement at age 12 can be attributed to genetic factors independent of tested achievement at age 10. The C_2_ estimate of .08 just reaches significance, indicating that for tested achievement there is some influence of shared environment on this index of added value.

### Current achievement independent of both previous achievement and ability

**Figure 3:**
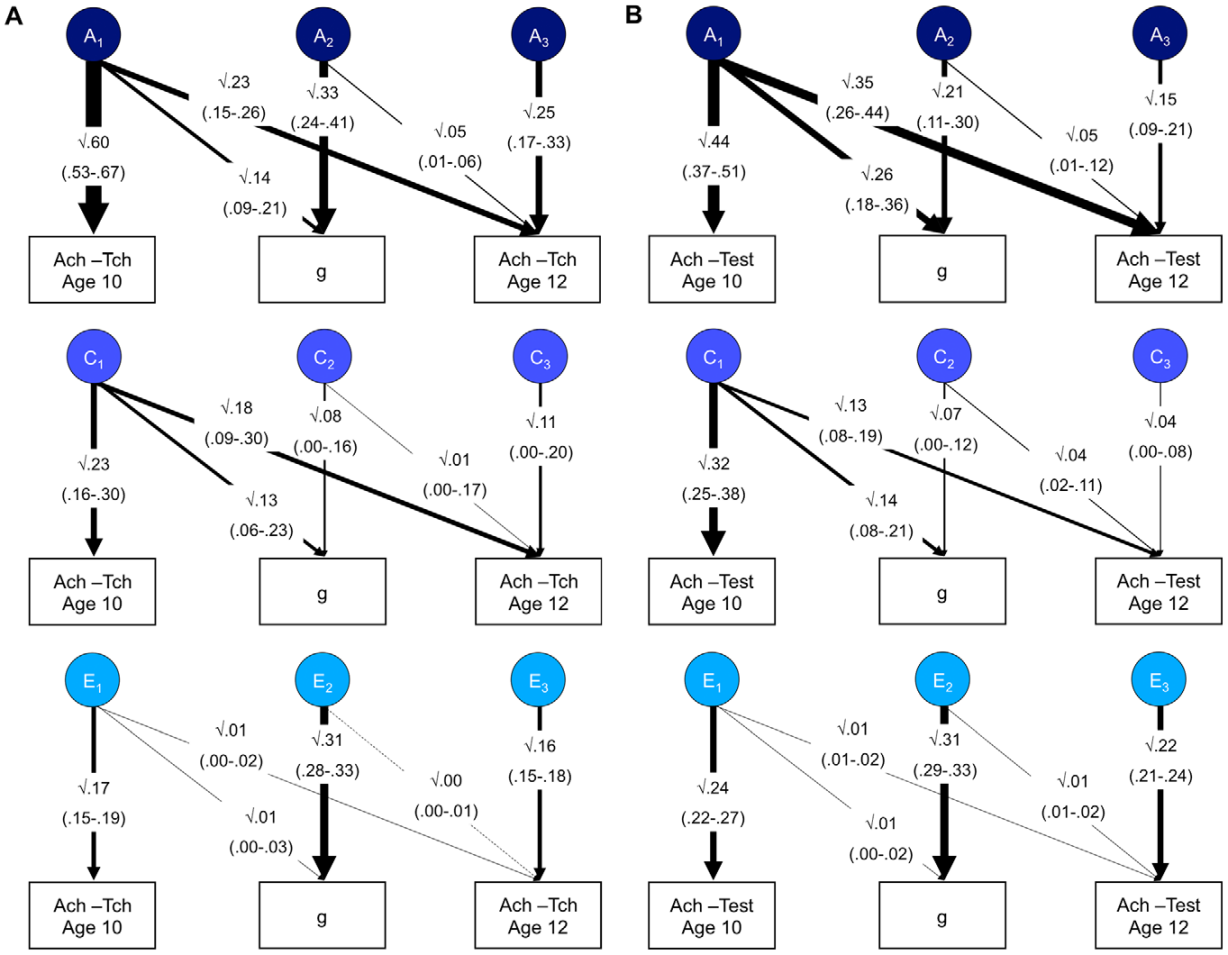
Trivariate analysis of 10-year achievement, general cognitive ability and 12-year achievement. Panel A =  Teacher-rated achievement. Panel B =  Test achievement. g =  general cognitive ability; Ach-Tch  =  Teacher-rated achievement; Ach-Test  =  test achievement; A =  additive genetic; C =  Shared environment; E =  Non-shared environment. The figures represent the results from a standardized Cholesky decomposition of twin data. 95% confidence intervals of the path estimates are shown in parentheses. The first factors assess genetic (A_1_), shared (C_1_) and non-shared environmental (E_1_) influences on 10-year achievement, some of which may also influence g and 12-year achievement. The second factor estimates influences on 12-year g that are independent of the influences on 10-year achievement, and which may also influence 12-year achievement (A_2_, C_2_ and E_2_). The third factor (A_3_, C_3_ and E_3_) estimates influences on 12-year achievement that are independent of those on 10-year achievement and 12-year g. Results indicate significant residual genetic influence on school achievement, even when the genetic and environmental co-variance with previous achievement and general cognitive ability has been removed (see the A_3_ path estimates).

Our results show that added value indexed by ability and by previous achievement continues to show genetic influence. What is the effect of creating an index of added value that corrects for *both* ability and previous achievement? We conducted an additional analysis that controlled for *both* previous achievement and g. We used a trivariate Cholesky decomposition model to assess the independent influences on 12-year achievement after controlling for the influences from both previous achievement and g. As shown in **Figure 3**, the results are similar to the averaged results shown previously when separately correcting for g (**Figure 1**) and for previous achievement (**Figure 2**). **Figure 3a**, which focuses on teacher-rated achievement at age 12, shows that residual genetic influence on 12-year achievement is significant (.25), whereas residual shared environmental influence is not significant (.11). Re-standardizing these residual estimates results in a heritability estimate of 48% (.25/(.25+ .11+ .16) = .48) for 12-year teacher-rated achievement corrected for both 10-year achievement and g; the re-standardized estimate of shared environment is 21% (.11/(.25+ .11+ .16) = .21). As shown in **Figure 3b**, results were also similar for adjusted test achievement: significant residual genetic influence (.15) and non-significant residual shared environmental influence (.04). Re-standardized heritability was 37% (.15/(.15+ .04+ .22) = .37) and re-standardized shared environment was 10% (.04/(.15+ .04+ .22) = .10).

Note that because these analyses require three composite measures at two different ages we focus on the model-fitting results, rather than the multiple regression, because the structural equation model does not require all of the measurements to be non-missing and therefore can use all available data. Nonetheless, results from the multiple regression, based on a smaller sample (just 334 pairs for teacher-rated achievement and 1646 pairs for test achievement), were highly similar, with twin correlations of 0.52 and 0.35 for MZ and DZ twins for teacher-rated achievement, and 0.47 and 0.27 for MZ and DZ twins for test performance.

## Discussion

The purpose of this study was to test the hypotheses that added value indices of school achievement are less influenced by genetic factors and more influenced by shared (school) environmental factors than raw achievement scores. We find that neither nature nor nurture support these predictions about added value.

### Achievement independent of attainment: Nature

Although other genetic studies have shown that achievement and *g* are linked genetically [10], [13], [18], [41], [42], we believe this is the first report of genetic and environmental influences on *g-*free achievement scores, as well as the first report of genetic and environmental influences on current achievement that is independent of previous achievement. For teacher assessments, heritability for *g*-free achievement is 0.49, which is almost as great as the heritability of 0.52 for achievement uncorrected for *g*. For test scores, *g-*free achievement was also significantly and substantially heritable (0.40) although non-significantly less heritable than achievement uncorrected for *g* (0.55).

The pattern of results is similar even if we use prior achievement as a covariate, because not only do brighter children perform better at previous measurement occasions, they also learn more subsequently. Most importantly in the present context, we find that this index of added value is just as heritable as raw achievement scores and *g-*free achievement scores. The heritability estimates for 12-year achievement independent of 10-year achievement are 0.47 for teacher assessments and 0.46 for test scores. In other words, current achievement independent of previous achievement shows just as much genetic influence as raw achievement. A more elegant way to address this question uses multivariate genetic analysis, which comes to the same conclusion (**Figures 1** and **2**).

Given that g and previous achievement are both important predictors of current achievement, we also conducted similar analyses where we controlled for both g and previous achievement in the same model (**Figure 3**). The results were striking, indicating that even when previous achievement and a child's general cognitive ability are both removed, the residual achievement measure is still significantly influenced by genetic factors (heritabilities of 48% and 37% respectively for teacher-ratings and test data). The main point, to which we shall return, is that corrected-achievement scores are influenced by genetic factors that are independent of those influencing *g* or previous achievement. In other words, if we were to identify specific genes associated with *g*, as is beginning to happen [43], [44], [45], these genes would not be associated with *g*-free achievement scores. Stated more positively, these results indicate that we could find genes associated with achievement independent of *g.*

### Achievement independent of attainment: Nurture

Finding that the heritability of corrected-achievement is about 50 percent implies that about half of the reliable variance is due to environmental differences between children; the twin method is equally effective at demonstrating the effects of nurture as of nature. The twin method is able to carry the analysis of nurture a step further, and distinguish two types of environmental influence: *shared environment* that contributes to the similarity of siblings growing up in the same family and attending the same school and *non-shared environment* which does not [7]. In our study, shared environmental influence accounts for 30 percent of the variance of teacher-rated achievement and 20 percent of the variance of achievement test scores. Although it would be reasonable to expect that shared environment plays a larger role in corrected-achievement, we show for the first time that shared environment has even less of an effect here. For example, shared environment accounts for only 21 percent of the variance of *g-*free teacher-rated achievement and only 6 percent of the variance of *g-*free achievement test scores.

An environmental finding in need of further detailed investigation involves non-shared environment. Controlling for ability or previous achievement removed a large proportion of the shared environmental influence in this sample, particularly for the test measures of achievement, but it did not remove the influence of the non-shared environment. This resulted in a larger proportion of the corrected-achievement scores being attributed to non-shared environmental factors. As with all measures, some of this influence may be measurement error.

We believe that understanding these non-shared environmental influences will be key in identifying the truly environmental influences on school achievement. The twin method provides substantial evidence for the important role of non-shared environmental influences on a range of cognitive and behavioural measures [7], but what are these non-shared environmental influences? In the twin design, non-shared environmental influences are those that do not contribute to similarity between twins, and which are conceptualized as individual-specific environments. A classic example of a non-shared environment is that of an accident or illness experienced by one twin and not the other [46]. Other examples include having different teachers, having different friends and participating in different extra-curricular activities. The current results highlight the major contribution that non-shared environmental factors make on school achievement – independent of g and independent of earlier achievement. These influences account for a large proportion of the variance in g-free achievement scores, 54% of the variance in the case of g-free test achievement.

A powerful way of identifying what these non-shared environmental factors are is to study differences within pairs of monozygotic (identical) twins, because the only factor that makes members of an identical twin pair different from one another is the non-shared environment. Therefore it is possible to correlate the differences in achievement scores within pairs of identical twins with differences in environmental exposure to determine whether such environmental exposures account for this non-shared environmental influence [47]. Such analyses have been carried out for some educationally relevant environments and school achievement measures [47], [48]. In a recent pilot study we found that differences within pairs of identical twins in classroom experiences were associated with differences in performance and behavior [49]. For example, differences in positivity about school and differences in flow (a measure of how focused a person is on the task at hand, or ‘in the zone’) were associated with differences in performance in mathematics and science respectively. We intend to follow-up this pilot with a large and comprehensive investigation of the non-shared environmental influences that impact on adolescent school performance. The present results suggest that the search for specific non-shared environmental influences will be facilitated by the use of achievement scores free of g and free of prior achievement – because these residualized achievement scores show less shared environmental influence and more non-shared environmental influence.

### The future of added value

Despite the reasonableness of assuming – or hoping – that a measure of achievement independent of previous attainment (either g or previous school achievement) could be constructed that would be a pure measure of the school or learning environment, about half of the variance of corrected-school achievement is due to genetic differences between children. This does not mean that the enterprise has failed, or is hopeless. There is no doubt that these indices of knowledge gain are useful at an individual level. However, averaged out at the school level, they do not necessarily provide a pure indication of the school's effectiveness [50].

Even though corrected-school achievement is not free of genetic influence, it is at least free of genetically-driven attainment as indexed by either *g* or by previous achievement, which makes it a useful step towards assessing the value that schools add to children's achievement. What is new from our study is the finding that what is left is still heritable. In addition, we have shown that contrary to expectations, indices of added value show only minimal shared environmental contributions.

Because half of the variance of achievement is not heritable, it would seem theoretically possible to create a better index of schools' added value by correcting for other genetically influenced traits. What are these other genetically influenced factors that affect children's achievement at school? Possible candidates include specific cognitive abilities not tapped by *g*, personality, interests, attitudes, motivation, and even psychopathology and health, all of which are at least moderately heritable [7]. For example, we have recently shown that self-perceived ability is genetically related to school achievement independent of *g* [51]. However, even after removing the genetic variance of both self-perceived ability and *g*, achievement scores are still substantially heritable.

The concept of added value is not used in all educational systems; however, these results are relevant to any educational system because they highlight the dynamic influence of genes and environments on school-related processes. For example, it seems a reasonable assumption that genetic influences are the same throughout development because children's DNA sequences do not change. However, these results suggest that new genetic influences can come into play even between 10 and 12 years of age because there are genetic influences specific to 12-year achievement that did not influence achievement at 10 years. In contrast, unlike DNA sequence, we know that environments change during development – even the classroom a child finds themselves in changes from year to year, but what these results suggest is that we should be focusing on changes in non-shared (as opposed to shared) environments. That is, environmental influences that affect added value are unique to individuals.

If we take seriously these findings that demonstrate that all aspects of achievement are suffused with genetic influence, we must conclude that it may not be possible to devise a measure of achievement that reflects only environmental influences such as the added value of schools. What follows from such a conclusion? What should *not* follow is the nihilistic notion that if achievement shows genetic influence there is nothing that can be done about it. Heritability does not imply immutability [7]. Nonetheless, the pervasiveness of genetic differences among children suggests the need to re-examine the role of education. Instead of thinking about education as a way of countering genetic differences among children, the field of education might profit from accepting that children differ genetically in *how* and *how much* they learn. This way of thinking is compatible with the current trend towards personalizing education by optimizing children's learning [52], which is increasingly possible through the use of interactive information technology. The opposite of personalized education is the attempt to use education to equalize children's learning, which if successful would have an unintended consequence: The resulting differences between children in their achievement would be even more heritable because one source of environmental variation would be eliminated.

More generally, instead of thinking about education as *instruction* (from the Latin *instruo*, which means ‘to build in’), this genetic perspective on learning suggests a return to the original meaning of *education* (from the Latin *educatio,* which means ‘to draw out’). That is, instead of a model of instruction in which children are the passive recipients of knowledge, a genetically sensitive approach to education suggests an active view of learning in which children select, modify and create their own education in part on the basis of their genetic propensities. In genetics, such processes are called *genotype-environment correlation* [53]. A correlation between an individual's genes and their specific environments would be estimated as part of the genetic (A) component in the twin design.

Genotype-environment correlation offers new ways of thinking about the environmental interface in which genotypes become phenotypes. Specifically, achievement independent of ability may be just as heritable as achievement including ability because achievement is as much a function of genetically-driven appetites as of aptitudes. Genotype-environment correlation is a way in which children add value to their own environments. Effective, responsive education systems may well be ones that increase the magnitude of such correlations between nature and nurture.
